# Transposon insertion libraries for the characterization of mutants from the kiwifruit pathogen *Pseudomonas syringae* pv. actinidiae

**DOI:** 10.1371/journal.pone.0172790

**Published:** 2017-03-01

**Authors:** Carl H. Mesarich, Jonathan Rees-George, Paul P. Gardner, Fatemeh Ashari Ghomi, Monica L. Gerth, Mark T. Andersen, Erik H. A. Rikkerink, Peter C. Fineran, Matthew D. Templeton

**Affiliations:** 1 Bioprotection Portfolio, The New Zealand Institute for Plant & Food Research Limited, Auckland, New Zealand; 2 Laboratory of Molecular Plant Pathology, Institute of Agriculture and Environment, Massey University, Palmerston North, New Zealand; 3 Bio-Protection Research Centre, New Zealand; 4 School of Biological Sciences, University of Canterbury, Christchurch, New Zealand; 5 Department of Biochemistry, University of Otago, Dunedin, New Zealand; 6 Department of Microbiology and Immunology, University of Otago, Dunedin, New Zealand; 7 School of Biological Sciences, University of Auckland, Auckland, New Zealand; University of Helsinki, FINLAND

## Abstract

*Pseudomonas syringae* pv. actinidiae (*Psa*), the causal agent of kiwifruit canker, is one of the most devastating plant diseases of recent times. We have generated two mini-Tn*5*-based random insertion libraries of *Psa* ICMP 18884. The first, a ‘phenotype of interest’ (POI) library, consists of 10,368 independent mutants gridded into 96-well plates. By replica plating onto selective media, the POI library was successfully screened for auxotrophic and motility mutants. Lipopolysaccharide (LPS) biosynthesis mutants with ‘Fuzzy-Spreader’-like morphologies were also identified through a visual screen. The second, a ‘mutant of interest’ (MOI) library, comprises around 96,000 independent mutants, also stored in 96-well plates, with approximately 200 individuals per well. The MOI library was sequenced on the Illumina MiSeq platform using Transposon-Directed Insertion site Sequencing (TraDIS) to map insertion sites onto the *Psa* genome. A grid-based PCR method was developed to recover individual mutants, and using this strategy, the MOI library was successfully screened for a putative LPS mutant not identified in the visual screen. The *Psa* chromosome and plasmid had 24,031 and 1,236 independent insertion events respectively, giving insertion frequencies of 3.65 and 16.6 per kb respectively. These data suggest that the MOI library is near saturation, with the theoretical probability of finding an insert in any one chromosomal gene estimated to be 97.5%. However, only 47% of chromosomal genes had insertions. This surprisingly low rate cannot be solely explained by the lack of insertions in essential genes, which would be expected to be around 5%. Strikingly, many accessory genes, including most of those encoding type III effectors, lacked insertions. In contrast, 94% of genes on the *Psa* plasmid had insertions, including for example, the type III effector *HopAU1*. These results suggest that some chromosomal sites are rendered inaccessible to transposon insertion, either by DNA-binding proteins or by the architecture of the nucleoid.

## Introduction

*Pseudomonas syringae* is a highly successful species complex that comprises important plant pathogens and epiphytes, and has recently been found in non-agricultural environments such as waterways [1, 2]. Pathogenic isolates of *P*. *syringae* usually have a narrow host range and have been grouped into a complex of approximately 60 pathovars, ten of which are now recognized as separate species [3]. Differences in host specificity are a function of the specific effector and secondary metabolite profiles they possess in their accessory genomes. The kiwifruit (*Actinidia* spp.) vine disease pathogen *P*. *syringae* pv. actinidiae (*Psa*) was first formally identified in Japan in 1984 [4, 5], and was subsequently found in Korea in the 1990s [6]. Both these strains caused canker symptoms, but did not spread from their country of origin. In 2008, a particularly virulent canker-causing strain of *Psa* was reported in Italy and it quickly decimated plantings of *A*. *chinensis* var. *chinensis* cultivars, particularly ‘Hort16A’, ‘Hongyang’ and ‘Jin Tao’. This strain was later found in other major kiwifruit growing regions of New Zealand and Chile in 2010 [7]. Phylogenetic analysis of the core genomes from 25 isolates of *Psa* representing isolates from all locations where *Psa* had been reported, indicated that the canker-causing isolates formed three clades, which were subsequently equated to biovars [8, 9]. The first biovar comprised the initial isolates from Japan, the second contained those collected in the 1990s from Korea, and the third consisted of the outbreak isolates from Italy, New Zealand, Chile and China. Isolates within biovar 3 differed by very few single nucleotide polymorphisms (SNPs), suggesting that this was a clonal population [8, 10, 11]. The isolates from New Zealand, Italy, Chile and China possessed different members of a family of integrative conjugative elements [8, 10, 11]. More recently, another canker-causing biovar of *Psa* has been found in Japan, which appears to be most closely related to biovar 2 from Korea [12]. Despite the relatively small number of SNPs in the core genomes of the canker-causing biovars, each has a surprisingly varied accessory genome, with different complements of genes encoding effectors and toxins [8, 13]. Many of these genes are coded on active mobile genetic elements [8, 10].

Transposon mutagenesis has long been used in bacterial genetics to identify and functionally characterize individual genes involved in pathogenicity. Transposon-based signature-tagged mutagenesis and genetic foot-printing strategies have identified essential genes and those involved in causing disease from a number of plant and human pathogens [14–16]. When combined with next generation sequencing technologies, the majority of transposon insertion events can be identified and mapped to a reference genome [17, 18].

In this paper we have generated two independent mini-Tn*5*-based transposon mutant libraries of *Psa* ICMP 18884. The first, a ‘phenotype of interest’ (POI) library, comprises 10,368 individually gridded mutants. The second, a ‘mutant of interest’ (MOI) library, consists of around 96,000 pooled mutants and has been sequenced on the Illumina MiSeq platform, with transposon insertion sites mapped onto the *Psa* ICMP 18884 genome [19]. We have demonstrated the utility of both libraries through the identification of several auxotrophic and motility mutants (POI library), as well as a mutant disrupted in a predicted component of the *Psa* lipopolysaccharide (LPS) biosynthesis pathway (MOI library). These libraries set the foundation for future studies aimed at dissecting the mechanisms of *Psa* growth and pathology.

## Materials and methods

### General materials and methods

The percentage of agar used in Lysogeny broth (LB), King’s B (KB) [20], and M9 minimal (M9) [21] media was 1.5% (w/v). The final concentration of kanamycin (Km) used in agar and liquid media was, unless otherwise stated, 50 μg/mL. *Escherichia coli* and *Psa* were routinely cultured at 37°C and room temperature (~20°C), respectively. All kits and reagents were used, except where specified, in accordance with the manufacturer’s instructions.

### Bacterial isolate used in this study

*Psa* ICMP 18884 (biovar 3; PRJNA71845; SAMN02727983), which was collected in 2010 from the kiwifruit cultivar *Actinidia chinensis* var. *deliciosa* ‘Hayward’ in Rangiuru, Te Puke, Bay of Plenty, New Zealand, was used in this study [8, 22]. From this point forward, *Psa* ICMP 18884 will be referred to as *Psa*.

### Plasmid DNA used for transposon mutagenesis of *Psa*

Plasmid DNA used for transposon mutagenesis of *Psa* was pKRCPN2 (S1 Fig), a derivative of pDS1028 [23, 24]. pKRCPN2 harbors the mini-Tn*5*-based transposon Tn-DS1028*uidA*Km, which itself carries a *uidA* β-glucuronidase (GUS) reporter gene and Km resistance cassette for the tracking and selection of *Psa* transformants, respectively (Kevin Roberts, unpublished). pKRCPN2 also carries a tetracycline (Tc) resistance cassette and an R6K*γ* origin of replication for propagation in *pir*-dependent strains of *E*. *coli*, as well as a hyperactive transposase for the transposition of Tn-DS1028*uidA*Km [25]. Briefly, for propagation, *E*. *coli* strain BW20767 was transformed with pKRCPN2 using a standard electroporation protocol, with selection overnight on LB agar medium containing 10 μg/mL Tc. A single colony was inoculated into 100 mL LB-Tc liquid medium and cultured overnight at 120 rpm. Plasmid DNA was purified using a PureLink HiPure Plasmid Midiprep Kit (Invitrogen). In addition to pKRCPN2, a second plasmid, pKRCPN1, carrying the mini-Tn*5*-based transposon Tn-DS1028*lacZ*Km [23], was trialed for transposon mutagenesis of *Psa*. pKRCPN1 DNA was propagated as described above for pKRCPN2.

### Transposon mutagenesis of *Psa*

*Psa* was stored at –80°C with 15% v/v glycerol as a cryoprotectant. When required, cells were streaked onto KB agar medium and cultured for 3 d. A single colony was inoculated into 50 mL KB liquid medium and cultured overnight at 120 rpm to an OD_600_ of 2–4. *Psa* was prepared for transformation by electroporation using a modified microcentrifuge-based protocol [26]. Briefly, 6 mL of liquid culture were distributed into four microcentrifuge tubes and cells collected by centrifugation at 17,000 × *g* for 30 s. The supernatant was removed, the cell pellet gently washed by re-suspension in 1 mL 300 mM sucrose, and the cells collected by centrifugation as above. This washing step was repeated, and the four cell pellets combined by gentle re-suspension in 100 μL 300 mM sucrose. For electroporation, 1 μg pKRCPN2 DNA in 10 μL sterile water was gently mixed with the competent cells and transferred to a 2-mm gap-width electroporation cuvette. Electroporation was carried out with pulse settings of 25 μF, 200 Ω and 2.5 Kv. Control cells were pulsed with sterile water in place of plasmid DNA. Immediately following electroporation, 1 mL KB liquid medium was added, and a tailored cell recovery regime initiated for the purpose of generating either an MOI or POI library (see below).

To generate the MOI library (S2 Fig), *Psa* cells from three independent electroporations, each in 1 mL KB liquid medium from above, were combined, added to a 50-mL tube containing 47 mL KB liquid medium, mixed by gentle inversion, and allowed to recover at 50 rpm for 1 h. Km was added, and 100 μL of the cell suspension dispensed into each well of five 2 mL 96-well round bottom polypropylene plates (Axygen) containing 900 μL KB-Km liquid medium. The number of mutants per well was estimated by plating a serial dilution of the cell suspension onto LB-Km agar medium. The 96-well plates were covered with gas-permeable rayon sealing tape (Nunc) and cultured without shaking for 3 d. Following this culturing step, cells were gently re-suspended by pipetting, and 250 μL from all wells of each column or row of the five 96-well plates were pooled and stored at –20°C for future DNA extraction (i.e. a total of 12 pooled column [A1–H1, A2–H2, A3–H3, A4–H4, A5–H5, A6–H6, A7–H7, A8–H8, A9–H9, A10–H10, A11–H11 and A12–H12] and eight pooled row [A1–A12, B1–B12, C1–C12, D1–D12, E1–E12, F1–F12, G1–G12 and H1–H12] samples per plate). Next, 500 μL 60% v/v glycerol was added to each well (24% v/v final concentration) and mixed as above. Plates were covered with polyolefin sealing tape (Nunc) and stored at –80°C.

To generate the POI library (S2 Fig), *Psa* cells from a single electroporation were recovered at 50 rpm for 1 h. Recovered cells were diluted eight-fold into KB-Km liquid medium, plated onto LB-Km agar medium, and cultured for 3 d. Plates were stored at 4°C for a maximum of 5 d post electroporation. A total of 10,368 independent transposon mutants were picked into 108 450-μL 96-well polypropylene conical bottom plates (Nunc) containing 225 μL KB-Km liquid medium, and cultured without shaking for 2 d. Following this culturing step, 75 μL 60% v/v glycerol was added (15% v/v final concentration) and mixed by gentle pipetting. Plates were covered with polyolefin sealing tape and stored at –80°C.

### GUS assay

To determine whether the GUS reporter in *Psa* transposon mutants was active, 96 independent mutants, representing a single 96-well plate from the POI library, were spotted (5 μL) onto LB-Km agar medium containing 100 μg/mL 5-Bromo-4-chloro-3-indolyl β-ᴅ-glucuronide (X-Glc), and cultured in the dark for 3 d.

### Arbitrary PCR experiments

To identify the specific transposon insertion sites within the chromosomal or plasmid DNA of *Psa* mutants, a two-step arbitrary polymerase chain reaction (PCR) experiment was performed [27–29] (see S1 Methods and primers listed in S1 Table). To extract total DNA, independent transposon mutants from the POI library grown on KB-Km or LB-Km agar medium were picked into microcentrifuge tubes containing 100 μL sterile water, and heated to 95°C for 10 min. Cell debris was then collected by centrifugation at 16,000 × *g* for 1 min, and 1 μL of supernatant used as a template for arbitrary PCR step 1. Amplicons from arbitrary PCR step 2 were resolved by gel electrophoresis in 1% w/v agarose, purified using a NucleoSpin Gel and PCR Clean-up Kit (Macherey-Nagel), and sequenced at Macrogen Inc. (Korea) using the PF1212 primer (S1 Table). To determine the position of transposon insertion sites, nucleotide sequences were mapped to the *Psa* chromosome or plasmid DNA sequence using BLASTn [30], and visualized using Geneious R9 software [31].

### Auxotroph screening

*Psa* mutants from eleven 96-well plates (1,056 individuals) of the POI library were replica-plated onto polystyrene OmniTrays (Nunc), the first containing M9-Km agar medium, and the second containing KB-Km agar medium. For this purpose, a hand-held 96-pin replicator tool (Nunc) was used, with the following sterilization protocol between library plates: 50% v/v bleach for 10 s, water for 15 s, 100% v/v ethanol for 10 s, and fan-dry for 30 s. All replica-plating was carried out in triplicate, with the OmniTrays cultured for 4 d. *Psa* mutants that grew only on KB-Km agar medium (complete auxotrophs), or strongly on KB-Km agar medium, but weakly on M9-Km agar medium (partial auxotrophs), were investigated further by arbitrary PCR and amplicon sequencing to identify the genes in these mutants disrupted by the transposon.

### Motility screening

*Psa* mutants from eleven 96-well plates (1,056 individuals) of the POI library were inoculated into 450-μL 96-well polypropylene conical bottom plates containing 300 μL tryptone broth-Km medium (1% w/v tryptone, 0.5% w/v NaCl) using the 96-pin replicator tool, and cultured without shaking for 2 d. From these starter cultures, the mutants were inoculated as above into new 450-μL 96-well polypropylene conical bottom plates containing 300 μL tryptone broth-Km, and incubated as above overnight. Using the replicator tool, the mutants were then replica-plated in triplicate onto polystyrene OmniTrays, the first containing KB agar medium, and the second containing swimming agar medium (1% w/v tryptone, 0.5% w/v NaCl, 0.3% w/v agar), both supplemented with 25 μg/mL Km. Plates were cultured for 4 d. Mutants identified as non-swimmers (no swimming ring), poor-swimmers (minimal swimming ring), and super-swimmers (enlarged swimming ring) were investigated further by arbitrary PCR and amplicon sequencing to identify the genes in these mutants disrupted by the transposon. It is worth noting that mutants on the plate boundaries, including those at positions A1–A12, H1–H12, A1–H1 and A12–H12, which are surrounded by five rather than eight other mutants, were not considered in this experiment. It was reasoned that these mutants could be incorrectly identified as super-swimmers, since they likely experience fewer density-dependent restrictions on colony growth.

### Colony morphology screening

*Psa* mutants with non-wild-type growth morphologies on LB-Km agar medium were identified by eye and under a Leica MZ12 stereoscopic microscope (Leica Microsystems). Arbitrary PCR, followed by amplicon sequencing, was then used to determine the specific transposon insertion sites within these mutants. Colony images were acquired with the Leica MZ12 stereoscopic microscope equipped with a Leica DFC 320 R2 digital camera. Images were processed using the Leica application suite v3.8.0. For storage, mutants were inoculated into 3 mL LB-Km liquid medium, and cultured overnight at 120 rpm. Following the addition of glycerol (15% v/v final volume), mutants were stored at –80°C.

### Sequencing of the MOI library

For sequencing of the MOI library, a total of 60 pooled column and 40 pooled row samples, representing the entire library, were first subjected to total DNA extraction. The 100 samples, which were previously stored at –20°C (see above), were thawed and transferred to microcentrifuge tubes on ice. Cells were collected by centrifugation at 17,000 × *g* for 30 s, and the supernatant discarded. Total DNA was extracted using a Gentra Puregene Yeast/Bact. Kit for Gram-negative bacteria (Qiagen), but with the following modifications: (1) twice the recommended volume of Cell Lysis Solution, RNase A Solution, Protein Precipitation Solution, isopropanol, and ethanol was used; and (2) precipitated protein was collected by centrifugation for 8 min. From each of the DNA samples, 50 ng was combined in a single microcentrifuge tube (5 μg total), and the total volume made up to 50 μL using DNA Hydration Solution. With the exception of library amplification and purification (see below), the combined sample was then prepared for sequencing on the Illumina MiSeq platform by New Zealand Genomics Limited (NZGL; Centre for Genomics, Proteomics and Metabolomics, University of Auckland, New Zealand). DNA (5 μg) was fragmented to 300 bp using an M220 focused-ultrasonicator (Covaris). An Illumina TruSeq Nano library was then created with ~1 μg input DNA using a Tru-Seq DNA Low-Throughput (LT) PCR-Free Library Kit (Illumina). Library DNA was resolved by gel electrophoresis in 6% w/v polyacrylamide, and a band of ~500 bp excised and purified. A quality control assessment of the library was performed using a 2100 BioAnalyzer with a BioAnalyzer High Sensitivity (HS) DNA Analysis Kit (Agilent Technologies). Library amplification was carried out using JumpStart *Taq* DNA Polymerase (Sigma-Aldrich) with the Nextera_i5_Adapt_Tn-specific_5F/Cust_Illumina_Nextera_i7_Adap_Truseq_i5_Adap_V3.3_R primer pair (S1 Table), as per a previously published protocol [17]. PCR products were purified using the Agencourt AMPure XP PCR Purification System (Beckman Coulter), with elution in 30 μL sterile water. A second round of library quality control assessment was carried out by NZGL using both a BioAnalyzer HS DNA Analysis Kit and a Qubit dsDNA High Sensitivity (HS) Assay Kit (Thermo Fisher Scientific). Purified PCR products were sequenced on the Illumina MiSeq platform in a single 150-bp single-end run using the nested Cust_Seq_Tn-specific_5F primer (S1 Table).

### Bioinformatic analysis of the MOI library

Illumina MiSeq reads were processed with AdapterRemoval (v1.5.4) [32] and prinseq-lite (v0.20.4), using the parameters ‘-fastq stdin -out_good stdout -out_bad null -min_len 30 -trim_tail_right 2 -trim_ns_right 1 -trim_qual_window 3 -trim_qual_right 30’ [33]. The resulting reads were mapped to the *Psa* genome [19] using the Bio::TraDIS toolkit [34], with parameters selected to increase the specificity (i.e. reduced false-positive rate) of the outputs. The command-line parameters used for this were: ‘bacteria_tradis -f fastqfiles -t 'ATAAATCTAGAGTCGACCTGCAGGCATGCAAGCTTCAGGGTTGAGATGTGTATAAGAGACAG' -r Psa-ICMP-reference-genome.fa—smalt_s 1—smalt_k 20—smalt_r 0—smalt_y 1.0 -mm 0 -v’. In brief, the toolkit trimmed the Tn-DS1028*uidA*Km transposon tags from reads in the input fastq files (the tag is specified with the “-t” parameter), and the remaining trimmed sequences were mapped to the *Psa* genome using the SMALT short read mapper [35]. SMALT indexed the *Psa* genome into words of length 20 (—smalt_k 20), with a step size of 1 (—smalt_s 1). A minimum identity threshold of 1.0 for each mapped read was used (—smalt_y 1.0), and the number of allowed mismatches was set to 0 (-mm 0). The tabular results were converted to the GFF file format and visualised in either Artemis [36] or Geneious [31]. Illumina MiSeq reads were deposited in the Sequence Read Archive (SRA) at the National Center for Biotechnology Information (NCBI) under BioProject and BioSample IDs PRJNA71845 and SAMN02727983, respectively.

### Validation of the MOI library

To validate the MOI library, a mutant carrying a disruption in the *IYO_023025* gene was targeted for identification and retrieval using a PCR screen, similar to that described by Holeva *et al*. [37]. The DNA from pooled column and row samples was screened for disruptions in the *IYO_023025* gene by PCR using the PF1212/Common_LPS_R6 primer pair (S1 Table). PCRs were carried out with Platinum *Taq* DNA polymerase (Invitrogen), and in a final volume of 20 μL containing 20 ng pooled DNA template. PCR products were resolved by gel electrophoresis in 1% w/v agarose. Amplicons common to specific pooled column and row samples were purified and sequenced using the PF1212 primer to confirm transposon-mediated disruption of the *IYO_023025* gene at the same site. Pooled column and row samples that gave amplicons with identical transposon insertion sites were used as coordinates to identify intersecting wells containing the *IYO_023025* mutant. An aliquot of the identified well was diluted 3:100,000 into LB-Km liquid medium, mixed by inversion, plated (1 mL) onto large square culture dishes (Nunc) containing LB-Km agar medium, and cultured for 3 d. Independent colonies were picked into 2-mL 96-well round bottom polypropylene plates containing 235 μL KB-Km liquid medium, and cultured for 3 d. To identify columns and rows containing the *IYO_023025* mutant, a second round of PCR screening was performed. Five-μL aliquots from each well of a given column or row were pooled into a PCR tube and the volume topped up to 100 μL with sterile water. At the same time, 75 μL 60% glycerol was added to each well of the four plates (15% v/v final concentration), mixed by gentle pipetting, covered with polyolefin sealing tape, and stored at –80°C. A crude DNA extraction, as described in the arbitrary PCR section, was performed on each pooled sample. Following extraction, 1 μL crude DNA extract was used as a template for PCR with the above-mentioned primers, and the products resolved by gel electrophoresis in 1% w/v agarose. Again, pooled column and row samples that gave amplicons with identical transposon insertion sites were used as coordinates to identify a shortlist of intersecting wells potentially containing the *IYO_023025* mutant. Next, to determine which of the shortlisted wells contained the mutant, the 96-well plates were thawed, and 5 μL from each well of all positive column samples in the plate was inoculated into independent microcentrifuge tubes containing 1 mL KB-Km liquid medium, and cultured overnight at 120 rpm. A crude DNA extraction was performed as described above. A total of 1 μL crude DNA extract was used as a template for PCR with the above-mentioned primers. Amplicons from the positive wells were purified and confirmed as belonging to the *IYO_023025* mutant by sequencing. Positively identified mutants were cultured on LB-Km agar medium for 3 d. *IYO_023025* mutants were photographed as described above.

## Results and discussion

### Preliminary characterization of *Psa* mutants generated by transposon mutagenesis

As a starting point for transposon mutagenesis experiments involving *Psa*, the transformation efficiencies of two plasmids, pKRCPN1 and pKRCPN2, which carry the mini-Tn*5*-derived transposons Tn-DS1028*uidA*Km and Tn-DS1028*lacZ*Km, respectively [23], were tested by electroporation. For pKRCPN1, fewer than five transformants/μg of DNA were consistently generated, while for pKRCPN2, this number routinely exceeded 25,000/μg. This observed discrepancy in transformation efficiency may have been due to the absence of restriction sites in pKRCPN2 DNA specific for the type I restriction modification system present in *Psa* [19]. Therefore, the use of pKRCPN1 was not pursued further.

**Fig 1:**
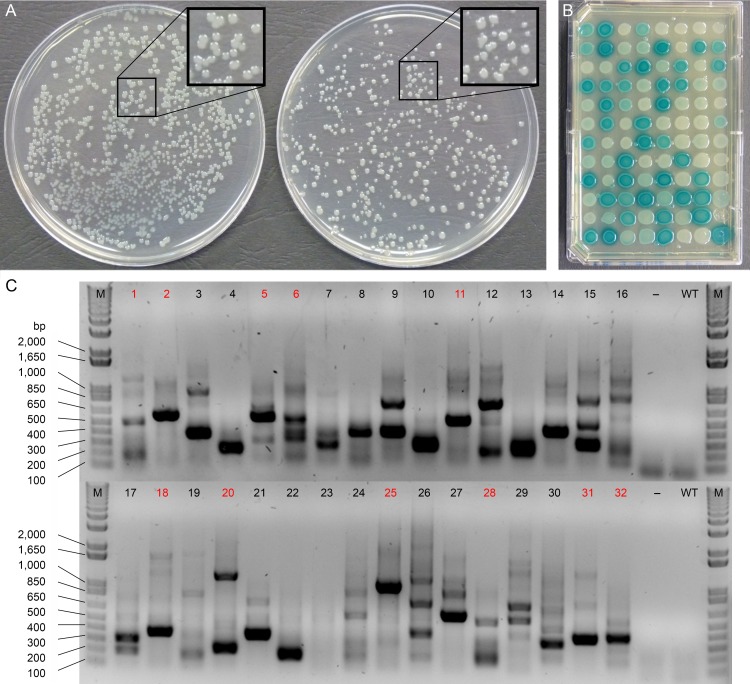
Preliminary characterization of *Psa* transposon mutants. **(A)** Colony size variation between wild-type *Psa* (left) and transposon mutants (right). A region of each plate, boxed in black, is enlarged (2×) for comparison; **(B)** Evaluation of the ability of transposon mutants to express GUS on KB-Km agar medium containing X-Gluc; **(C)** Arbitrary PCR to amplify transposon insertion sites from the genomic DNA of 32 independent transposon mutants (1–32). PCR amplicons from samples labelled in red were sequenced to characterize the specific location(s) of genome insertion by the transposon. M = DNA ladder, bp = base pairs,– = H_2_O negative control, WT = wild-type *Psa* genomic DNA.

Several lines of evidence suggested that the Tn-DS1028*uidA*Km transposon was inserting randomly into the *Psa* genome. The first was based on colony size during *in vitro* growth on LB agar medium. Colonies of wild-type (WT) *Psa* formed following electroporation in the absence of pKRCPN2 DNA were mostly uniform in size, although density-dependent differences were observed (Fig 1A). In contrast, the transposon mutants displayed variation in colony size (Fig 1A), suggesting that these observed differences in impaired growth were due to differences in the site of genome insertion by the transposon.

The second line of evidence supporting random transposon insertion was based on the finding that 96 independent *Psa* mutants exhibited varied levels of GUS activity on KB-Km agar medium containing X-Gluc (Fig 1B). GUS is an enzyme encoded by the promoter-less *uidA* gene on the transposon (S1 Fig), and varied *uidA* expression is dependent upon the specific location of transposon insertion within the *Psa* genome.

The third line of evidence supporting random transposon insertion was based on the integration sites of 32 independent, randomly chosen, *Psa* mutants by arbitrary PCR [28]. In this experiment, no PCR amplicons were generated from the WT DNA and DNA-free negative controls (Fig 1C). However, distinct PCR amplicon profiles were produced from the genomic DNA samples of all 32 independent mutants (Fig 1C). Sequencing of PCR amplicons from 12 randomly selected mutants revealed that each mutant carried only one transposon insertion site, and that this insertion site was unique (S2 Table).

### Generation and validation of the POI library

A large library of mutants individually gridded into microtitre plates is an extremely useful tool for identifying phenotypes of interest associated with targeted functional screens [29, 38, 39]. To generate a phenotype of interest (POI) library, we gridded 10,368 randomly selected, independent *Psa* mutants into 96-well plates. The POI library was validated by screening two separate subsets of library plates, each comprising 1,056 mutants, on M9 agar medium and swimming agar medium for phenotypes associated with auxotrophy and altered motility, respectively. Seventeen putative complete or partial auxotrophic mutants, as well as eight mutants with potentially altered motility, were identified from these screens. A list of genes disrupted in these mutants, including the specific sites of transposon insertion, as determined by arbitrary PCR and amplicon sequencing, is shown in S2 Table.

**Fig 2:**
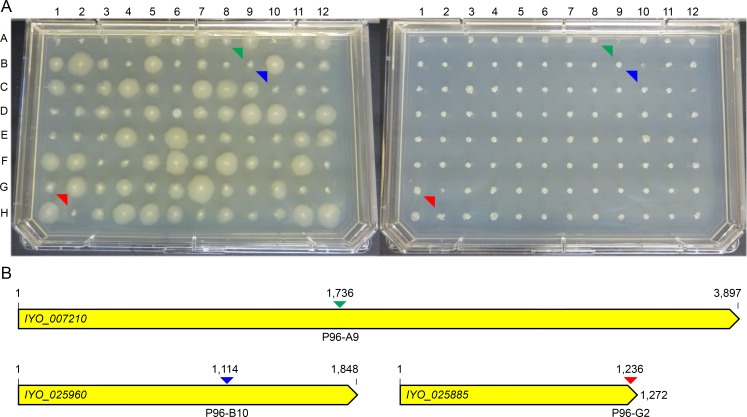
Screening the *Psa* phenotype of interest (POI) library for auxotrophic mutants. **(A)** Mutants were screened on KB agar medium (left) and M9 agar medium (right). Representative results for POI library plate 96 (P96) are shown. Auxotrophic mutants are denoted by green, blue and red arrows at positions P96-A9, P96-B10 and P96-G2, respectively; **(B)** Schematic of the *IYO_007210*, *IYO_025960* and *IYO_025885* genes disrupted in the auxotrophic mutants retrieved from positions P96-A9, P96-B10 and P96-G2, respectively in (A). Arrow colors correspond to the mutants highlighted in (A), and show the location of insertion by the transposon. All replica plating was carried out in triplicate.

A total of 13 genes were disrupted across the 17 putative auxotrophic mutants (S2 Table). An example of results obtained for the auxotrophic mutant screen is shown in Fig 2A, with a schematic of the disrupted genes shown in Fig 2B. Of the 13 genes identified, *IYO_007210*, *IYO_008330*, *IYO_008720* and *IYO_025960*, which putatively encode the phosphoribosylformylglycinamidine synthase enzyme PurL (purine biosynthesis), the quinolinate synthase enzyme NadA (NAD biosynthesis), the phosphoribosylglycinamide formyltransferase enzyme PurN (purine biosynthesis), and the dihydroxy-acid dehydratase enzyme IIvD (valine, leucine, isoleucine, pantothenate and coenzyme A [CoA] biosynthesis), respectively, were disrupted twice in independent locations (S2 Table), supporting an essential role for these genes in the growth of *Psa* on M9 agar medium. In line with this result, auxotrophic mutants with disruptions in homologs of at least three of these genes, generated using a similar mini-Tn*5*-based transposon mutagenesis approach, have been identified in other *Pseudomonas* species [29, 40].

The nine remaining genes (*IYO_001755*, *IYO_006345*, *IYO_006370*, *IYO_007710*, *IYO_008365*, *IYO_012780*, *IYO_025885*, *IYO_026305* and *IYO_027710*) were disrupted only once in the set of putative complete or partial auxotrophic mutants analyzed (S2 Table). Auxotrophic mutants in other *Pseudomonas* species with disruptions in homologs of at least two of these genes have been identified, including *IYO_012780* [29, 40] and *IYO_025885* [41, 42], which putatively encode the 3-isopropylmalate dehydratase enzyme LeuD (leucine biosynthesis) and the dihydroorotase enzyme PyrC (pyrimidine biosynthesis), respectively.

**Fig 3:**
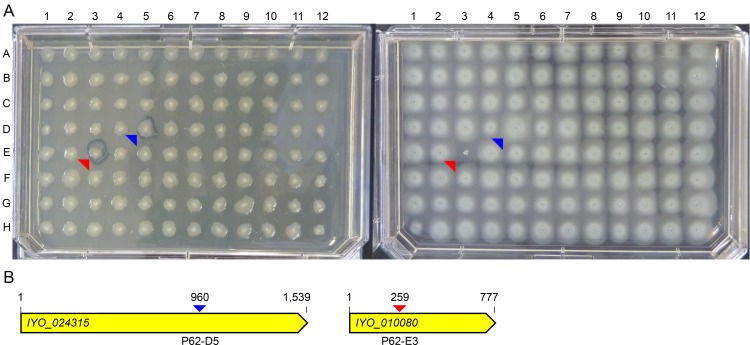
Screening the *Psa* phenotype of interest (POI) library for mutants with altered motility. **(A)** Mutants were screened on KB agar medium (left) and swimming agar medium (right). Representative results for POI library plate 62 (P62) are shown. Swimming mutants are denoted by a blue (super-swimming phenotype; position P62-D5) and red (non-swimming phenotype; position P62-E3) arrow. Note the significantly enlarged colony ring of the super-swimming mutant; **(B)** Schematic of the *IYO_024315* and *IYO_010080* genes, which are disrupted in motility mutants retrieved from positions P62-D5 and P62-E3, respectively in (A). Arrow colors correspond to the mutants highlighted in (A), and show the location of gene insertion by the transposon. All replica plating was carried out in triplicate.

To validate the POI library further and demonstrate its utility for mutant identification, we also screened for motility phenotypes in soft agar under nutrient-rich conditions. Swimming mutants are of particular interest, as motility has been shown for many plant pathogens to be critical for host invasion and virulence [43–45]. Furthermore, a large number of flagellar and chemotaxis genes have been identified in other bacteria, thus providing internal controls for our experiment. From these screens, four transposon mutants displayed a non-swimming phenotype (Fig 3A and S2 Table). Arbitrary PCR and amplicon sequencing revealed that all four disruptions occurred in genes known to be associated with the flagellar apparatus (i.e. *fliR*, *flhA*, *flgC* and *flgH*) [46]. Flagella are important in swimming motility, biofilm formation, and swarming motility [47], and disruptions in flagella-related genes have been identified in motility screens of transposon mutagenesis libraries from other *Pseudomonas* [15, 29].

Our motility screen also identified several genes which have not been previously linked to motility. Two mutants with 'super-swimming' phenotypes were identified with disruptions in the *IYO_020475* and *IYO_024315* genes, respectively (Fig 3A and S2 Table). *IYO_020475* encodes a homolog of PA3234, a probable sodium-solute symporter from *P*. *aeruginosa* PAO1 that has been linked to biofilm formation [48, 49], although a role in motility has not been previously reported. The other super-swimming mutant has a mutation in a gene (*IYO_024315*) that encodes a putative membrane protein TraG, which is required for F-pilus assembly [50], but again, a role in motility has not been previously identified. In addition to the super-swimmers, two weak-swimming mutants were identified that carry disruptions in the *IYO_007210* and *IYO_026350* genes, respectively (S2 Table). *IYO_026350* encodes a hypothetical transpeptidase-transglycosylase. The weak motility phenotype observed for this mutant could be due to impaired flagella, as these enzymes are involved in making gaps in the peptidoglycan layer for the insertion of the flagellar apparatus [51]. Notably, *IYO_026350* is co-located in the *Psa* genome with several flagella assembly genes. The other weak swimmer (P60-C4), which has a disruption in the *IYO_007210* gene, and was also identified as part of the auxotroph screen described above, putatively encodes PurL, a phosphoribosylformylglycinamidine synthase enzyme involved in purine biosynthesis. The basis for the motility defect of this mutant is unclear; however, purine biosynthesis genes have been linked to motility and biofilm defects in other bacteria [52–54].

**Fig 4:**
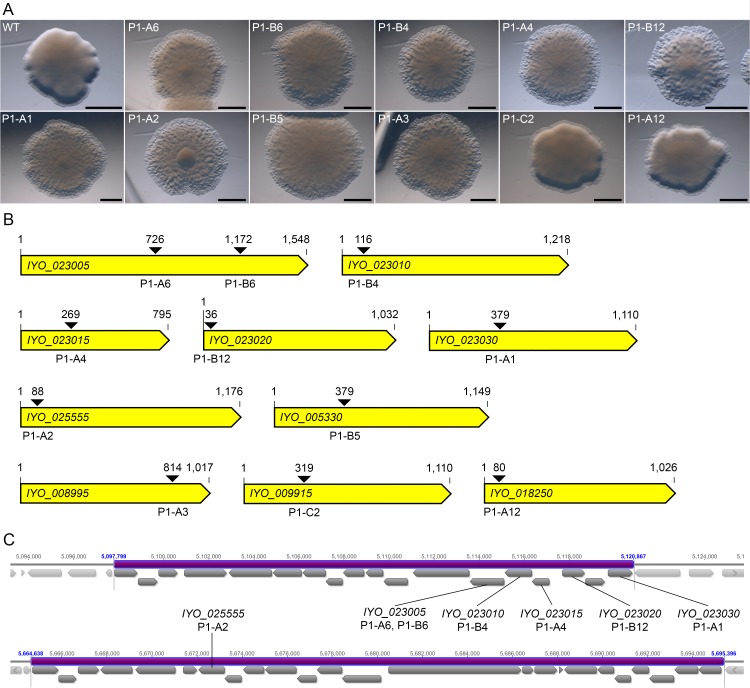
*Psa* transposon mutants with unusual colony morphologies. **(A)** Colony morphology of nine *Psa* mutants (P1-A6, P1-B6, P1-B4, P1-A4, P1-B12, P1-A1, P1-A2, P1-B5 and P1-A3). The colony morphologies of wild-type (WT) *Psa* and two transposon mutants with a WT-like phenotype (P1-C2 and P1-A12) are also shown. Bar = 2 μM; **(B)** Schematic of the genes disrupted in the transposon mutants of (A). Arrows show the location of gene insertion by the transposon; **(C)** Schematic of the predicted *Psa* common (top) and core (bottom) LPS biosynthesis pathway gene clusters (purple). The location of six genes from (B) within these clusters is shown.

A subset of *Psa* transposon mutants were screened for unusual colony morphologies by eye and under a stereoscopic microscope. WT colonies were smooth, domed, and with the exception of their relatively narrow borders, largely opaque (Fig 4A). In contrast, nine mutants were rippled, flat, and typically possessed a larger transparent border (Fig 4A). This colony morphology is similar to the ‘Fuzzy-Spreader’ (FS) mutant phenotype reported for *Pseudomonas fluorescens* SBW25. Such FSs have loss-of-function mutations in *pflu0478*, which encodes a glycosyl transferase that is predicted to modify LPS O antigens [55, 56]. Most *Psa* colony morphology mutants also demonstrated a more pronounced central mass of cells. This is best illustrated by colonies of the P1-A2 mutant (Fig 4A). Arbitrary PCR followed by amplicon sequencing revealed that six of the mutants had disruptions in near-consecutive genes of the predicted common LPS biosynthesis pathway gene cluster, while one mutant was disrupted for a gene in the predicted core LPS biosynthesis pathway gene cluster (Fig 4B and 4C and S2 Table). LPS is synthesized from at least three distinct macromolecules, including lipid A, which is membrane associated, as well as the core and common polysaccharides. Additional highly variable isolate-specific decoration on the outer surface of the molecule may also occur [57]. Interestingly, the two remaining mutants, like the LPS mutant P1-A1, both contained disruptions in genes that putatively encode glycosyl transferases (S2 Table). However, neither of these genes forms part of the predicted core and common LPS biosynthesis pathway gene clusters shown in Fig 4C. As a consequence, our visual screen may have identified additional, as yet unannotated, genes involved in LPS biosynthesis in other regions of the *Psa* genome.

### Generation and validation of the MOI library

As traditional gene knockout experiments in bacteria can be time-consuming (i.e. upwards of one month), we also generated a mutant of interest (MOI) library that can be used to recover desired mutants quickly. This library comprises around 96,000 independent mutants across five 96-well plates, with approximately 200 individuals per well. It has been indexed by taking genomic DNA samples from 60 pooled column and 40 pooled row samples (representing all columns and rows of the five 96-well plates), which can be screened by PCR to identify specific wells containing a mutant of interest. To catalogue the majority of transposon insertion sites (and thus mutants) within the library, it was sequenced on the Illumina MiSeq platform using a Transposon-Directed Insertion site Sequencing (TraDIS) approach [17, 18], with reads mapped to the *Psa* genome [19].

To demonstrate the utility of the MOI library, we set out to identify and retrieve a mutant disrupted in the *IYO_023025* gene, which putatively encodes a GDP-6-deoxy-ᴅ-lyxo-4-hexulose reductase enzyme of the *Psa* common LPS biosynthesis pathway (S2 Table), using a PCR screen. *IYO_023025* is located between the previously described LPS mutations in *IYO_023020* and *IYO_023030*, and is 897 bp in length (Fig 4C). Importantly, 30 mutants disrupted for the *IYO_023025* gene were identified in the MOI library by TraDIS read mapping, suggesting that the retrieval of such a mutant was possible. With this in mind, a primer pair comprising PF1212 (forward) and Common_LPS_R6 (reverse), which bind to the 3′ end of the transposon and 172 bp downstream of the *IYO_023025* stop codon, respectively, was designed for use in the PCR screen. Using this primer pair, it was determined that, to detect a disruption in the *IYO_023025* gene, the PCR amplicon size would need to be between 270 and 1,150 bp in length.

**Fig 5:**
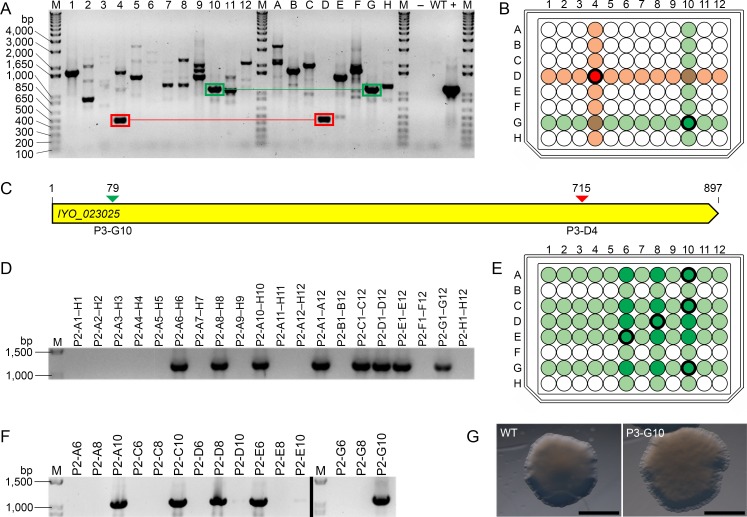
Validation of the *Psa* mutant of interest (MOI) library. **(A)** PCR screen to identify disruptions in the *IYO_023025* gene. Pooled genomic DNA samples from the columns (lanes 1–12) and rows (lanes A–H) of MOI library plate 3 (P3) were used as templates for PCR. Two sets of amplicons that share a specific *IYO_023025* disruption across a single pooled column and row sample are boxed in green and red, respectively; **(B)** Location of the P3-G10 and P3-D4 wells, which contain a mutant with an *IYO_023025* disruption specific to the PCR amplicons boxed in green and red in (A), respectively; **(C)** Schematic of the *IYO_023025* gene showing the location of transposon insertion sites identified in (A). Transposon insertion sites are denoted by arrows, and are color-coded to match the PCR amplicons boxed green and red in (A); **(D)** PCR screen to identify the *IYO_023025* disruption mutant from well P3-G10. Pooled genomic DNA samples from the columns and rows of a 96-well plate (P2) containing independent colony-forming units of well P3-G10 were used as templates for PCR; **(E)** Location of intersecting wells in P2 (dark green) that possibly contain the *IYO_023025* P3-G10 disruption mutant, as determined by the PCR amplicon profile in (D); **(F)** PCR screen to determine which of the intersecting wells in (E) contain the *IYO_023025* P3-G10 disruption mutant. Amplicons shown in the left and right panels (separated by a black line) are derived from different regions of the same gel. Wells that contain the mutant are shown in bold in (E); **(G)** Colony morphology of wild-type (WT) *Psa* and the *IYO_023025* P3-G10 disruption mutant. Bar = 2 μM, M = DNA ladder, bp = base pairs,– = H_2_O negative control, WT = WT *Psa* DNA.

PCR was carried out on the 12 pooled column and eight pooled row samples from each of the five 96-well plates (100 pooled samples total). The aim was to identify amplicons of appropriate size, as described above, and of apparent equal size between a specific pooled column and row sample of a given MOI library plate. In this way, matching amplicons, or rather, pooled samples that produced matching amplicons, could be used to provide coordinates for the identification of specific wells carrying a mutant disrupted in the *IYO_023025* gene. This is best illustrated in Fig 5, using MOI library plate 3 as an example. As shown in Fig 5A, several amplicons of appropriate size, and of apparent equal size between pooled column and row samples, were identified, including, for example, those boxed in green and red, respectively (Fig 5A). In these two cases, amplicon sequencing confirmed that both members of the pair corresponded to a disruption of the *IYO_023025* gene at the same location. In total, 20 PCR amplicons generated from the collection of five 96-well library plates, representing 10 matching pairs between pooled column and row samples, were selected for sequencing in this experiment. Nine of the 10 matching pairs were able to be confirmed, with only one amplicon not corresponding to a disrupted *IYO_023025* gene (S2 Table), highlighting the efficacy of this PCR screening methodology.

The pooled column and row samples corresponding to the matching amplicons boxed in green (row G, column 10) and red (row D, column 4) of MOI library plate 3 (Fig 5A) were used as coordinates to identify the intersecting wells P3-G10 and P3-D4 (Fig 5B), which, based on amplicon sequencing, contain a mutant with a disruption of the *IYO_023025* gene at position 79 and 715, respectively (Fig 5C). Since the former is a 5′ gene disruption (i.e. the disrupted gene is less likely to produce a fully or partially functional product), the mutant carrying this disruption was preferentially targeted for retrieval. For this purpose, 384 independent colonies from well P3-G10 were transferred to four 96-well plates for culturing and PCR screening. Here, all columns and rows were pooled (48 and 32 pooled samples, respectively), DNA was extracted, and the samples screened for the mutant of interest carrying a disruption at position 79 of the *IYO_023025* gene. As shown in Fig 5D, a total of three positive pooled column samples and five positive pooled row samples were identified in plate 2 (P2). This narrowed down the total number of possible positive wells in P2 that contained the mutant from 96 to 15 (positions A6, A8, A10, C6, C8, C10, D6, D8, D10, E6, E8, E10, G6, G8 and G10) (Fig 5E). DNA from all 15 wells was screened for the same disruption by PCR. A total of five wells (positions A10, C10, D8, E6 and G10) were positively identified as carrying the mutant disrupted for the *IYO_023025* gene (Fig 5F), and the disruptions were confirmed by amplicon sequencing. Unlike the FS-like colony morphology of the LPS mutants identified in the visual screen, the *IYO_023025* P3-G10 mutant had a phenotype much more like that of WT *Psa* and, for that matter, mutants P1-C2 and P1-A12 (compare Figs 4A and 5G). However, like the previously identified LPS mutants, P3-G10 was found to have a more rippled border than WT *Psa* (compare Figs 4A and 5G). This subtle phenotype probably explains why no mutants with a disruption in the *IYO_023025* gene were identified in the visual screen.

### Distribution of transposon insertion sites in the *Psa* genome

To determine the locations and frequencies of transposon insertion sites within the *Psa* chromosome and plasmid (which is single-copy), a conservative (or ‘specific’) TraDIS read mapping setting was used (S3 Table) [34]. Although the number of mapped insertions varied among this and another two settings (labelled ‘sensitive’ and ‘default’) (S3 Table), the subset of genes with insertions was identical in each case. This suggests that slight variations in sequence or alignment were responsible for differences in mapped insertions between the three settings. Importantly, all insertion sites determined experimentally through the sequencing of *IYO_023025* PCR amplicons from the MOI library (S2 Table) were also identified using the specific setting. In total, 24,031 and 1,236 transposon insertion sites were mapped onto the chromosome and plasmid, respectively, giving average insertion frequencies of 3.65 (chromosome) and 16.6 (plasmid) per kb (S3 Table). Assuming a random distribution of transposon insertions and an average length of 1 kb per gene, the probability of finding a particular chromosomal gene of interest disrupted by transposon insertion is 97.5%. This would indicate that the library is near-saturated. The insertion pattern over the chromosome was slightly biased toward the origin and tapered to the terminus (S3 Fig). This bias was also observed in *Salmonella*, and is most likely due to the use of exponentially growing cells for electroporation [58]. These cells possess multiple replication forks resulting in genes closer to the origin of replication having a greater copy number thus increasing the chance of an insertion event [58]. Avoiding this sort of bias might be achieved by using stationary-phase or starved cells. We did not observe a strong sequence-specific preference or bias towards AT-rich regions. In fact, insertion sites were ~2% more GC-rich than the background, in contrast to the Mariner transposon [59], sequence-logos of these regions showed no motif or strong sequence preferences for the transposon (data not shown). As expected, visual inspection of insertion sites mapped onto the *Psa* genome revealed genes with multiple insertions and those with none. In the example shown in S4A Fig, genes with a function in replication (DnaA, DNA polymerase and DNA gyrase) have no insertions, while those that function in a predicted type I restriction modification system complex and RecF have many insertions. One would clearly expect that those genes involved in replication are essential, while those involved in restriction modification are not.

A frequency distribution of the insertion index for all genes (i.e. the number of inserts per gene divided by gene length) should theoretically reveal a bimodal distribution, with genes essential to growth having no (or very few) insertions, while those not essential for growth should possess insertions [17]. While previous results indicated that the transposon was inserting randomly in the *Psa* genome, the frequency distribution of the insertion index for all genes did not reveal a bimodal distribution. Only 47% of chromosomal genes possessed inserts, which was far fewer than expected from the calculation of the likelihood of random insertion into a particular gene. Previous studies have indicated that less than 5% of bacterial genes are essential [58]. Strikingly, many accessory genes in the *Psa* chromosome, particularly those under the control of the HrpL regulon had very few insertions (S4B Fig). Furthermore, the genes of the HrpL regulon are dispersed throughout the chromosome and are not clustered around the replication terminus which has a lower insertion index (S3 Fig).

An explanation for this result might be that histone-like nucleoid structuring (H-NS) or related proteins are protecting specific regions in the genome from insertion. It was predicted that nucleoid or other DNA-binding proteins might bias TraDIS analysis [34], and recently this has been experimentally demonstrated for H-NS in *Vibrio cholerae* [60]. Notably, the *Psa* genome has at least three H-NS orthologues (*IYO_017020*, *IYO_021880* and *IYO_015055*). An alternative explanation may be that the topology or architecture of the chromosome sterically restricts transposon insertion [61]. It has recently been shown in the plant pathogen *Dickeya dadantii* that genes, present on different pathogenicity islands, involved in pectin degradation under the control of the KdgR regulon are potentially co-localized in an ‘archipelago’ [62]. Furthermore, the expression of these genes appears to be regulated by an H-NS orthologue [63].

In contrast to the chromosome pattern, the distribution of transposon insertion sites in the *Psa* plasmid showed less bias. Only five of the 78 predicted coding sequences had no inserts. One of these was a predicted antitoxin gene belonging to a RelE/ParE family plasmid stabilization system, which would be expected to be lethal. Significantly, *HopAU1* had 22.8 insertions per kb, which was by far the most of any *Psa* type III effector. The discrepancy between the insertion pattern of the chromosome and plasmid may reflect differences in topology and protein-binding characteristics of the DNA. Certainly, as the plasmid is single-copy, a greater insertion frequency in this DNA type cannot be explained by copy number.

## Conclusions

We have generated two mini-Tn*5*-based transposon mutant libraries of *Psa*. The utility of both libraries has been demonstrated through the discovery of new genes involved in motility (including those that give a super-swimming phenotype when disrupted), as well as in auxotrophy and LPS biosynthesis. The MOI library was predicted to be near saturation. However, 47% of chromosomal genes lacked insertions. Usually the number of essential genes represents about 5% of the genome. The observed discrepancy cannot be solely explained by the lack of insertions in essential genes, and surprisingly, many accessory genes such as those encoding type III effectors, which are clearly not essential for *in vitro* growth, lacked insertions. This may restrict the utility of these libraries for finding mutations in some genes, including those in pathogenicity. It has been demonstrated recently that H-NS proteins can protect genes from transposon insertion, as can the overall topology of the nucleoid. Strikingly, 94% of the plasmid genes, including the type III effector *HopAU1*, had multiple insertions. These results suggest that H-NS proteins and chromosome architecture might regulate the expression of the accessory genome in *Psa*.

## Supporting information

S1 Fig Vector map of pKRCPN2. pKRCPN2 harbors the mini-Tn*5*-based transposon Tn-DS1028*uidA*Km, which itself carries a *uidA* (β-glucuronidase; GUS) reporter gene and kanamycin resistance cassette (*kan2*) for the tracking and selection of *Psa* transposon mutants, respectively. pKRCPN2 also carries a tetracycline resistance gene (*TcR*) and an R6K*γ* origin of replication for propagation in *pir*-dependent strains of *Escherichia coli*, as well as a hyperactive transposase for transposition of Tn-DS1028*uidA*Km. Locations of the transposon-specific primers PF294, PF1212, Nextera_i5_Adapt_Tn-specific_5F and Cust_Seq_Tn-specific_5F are shown. The image was prepared using Geneious R9 software [31].
(TIF)

S2 Fig Flow diagram detailing generation of the *Psa* mutant of interest (MOI) and phenotype of interest (POI) libraries. The final concentration of kanamycin (Km) used in King’s B (KB) agar and liquid media was 50 μg/mL.
(TIF)

S3 Fig Distribution of transposon insertion sites across the *Psa* chromosome and plasmid. The scatter plots show the insertion index, computed for each protein-coding gene as a function of position on the *Psa* chromosome (left) and plasmid (right). In brief, the insertion index for each gene is computed using the count of unique transposon insertion sites in each gene, divided by the length of the gene. For example, a 108-bp length gene with 27 insertion sites has an insertion index of 27/108 = 0.25. A lowess curve (locally-weighted polynomial regression) is fitted to these two datasets, for the chromosome this shows a slight positional bias in the insertion indices. The genes near the origin of replication tend to have a higher insertion index, presumably because this region has a higher likelihood of receiving insertions during replication (two copies of the origin exist for longer than two copies of the terminus). The locations of *Psa* genes under the control of the HrpL regulon are shown by blue vertical dashes above the x axis.
(TIF)

S4 Fig Example of transposon insertion sites across the *Psa* chromosome. **(A)** a region of the *Psa* genome adjacent to the origin of replication; **(B)** a region from the conserved effector locus of the *Psa* genome. Transposon insertion sites are denoted by grey vertical lines, predicted coding regions as yellow bars and HrpL boxes by light blue triangles. The image was prepared using Geneious R9 software [31].
(TIF)

S1 Table Primers used in this study. (DOCX)

S2 Table List of *Psa* transposon mutants identified in this study. (DOCX)

S3 Table Transposon insertion settings and statistics. (DOCX)

S1 Methods (DOCX)
