# Supplementary figures and images for: Transposon insertion libraries for the characterization of mutants from the kiwifruit pathogen Pseudomonas syringae pv. actinidiae
Source: PLoS One. 2017 Mar 1;12(3):e0172790. doi: 10.1371/journal.pone.0172790 (PMC5332098; doi:10.1371/journal.pone.0172790)

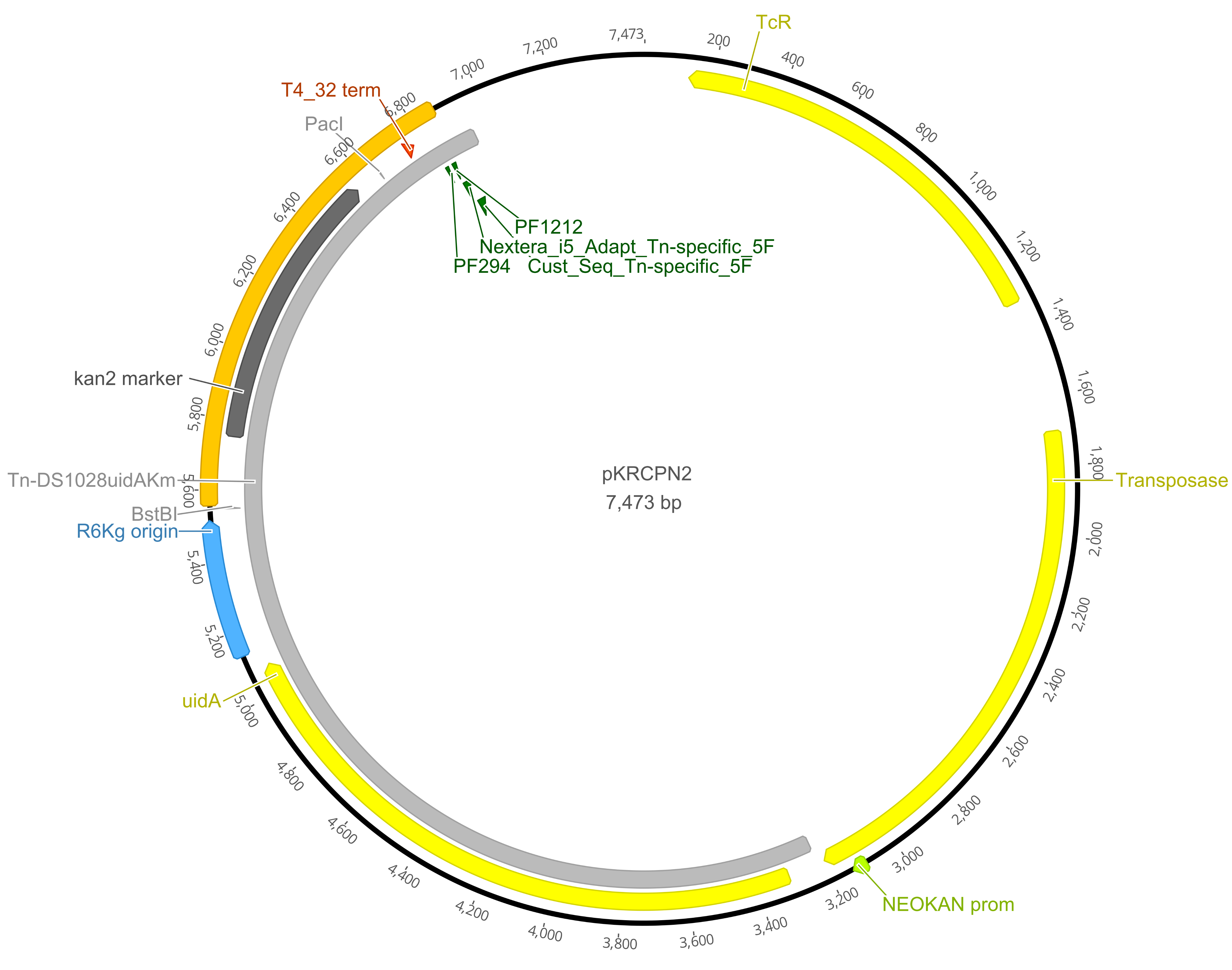

Supplement: S1 Fig — pKRCPN2 harbors the mini-Tn5-based transposon Tn-DS1028uidAKm, which itself carries a uidA (β-glucuronidase; GUS) reporter gene and kanamycin resistance cassette (kan2) for the tracking and selection of Psa transposon mutants, respectively. pKRCPN2 also carries a tetracycline resistance gene (TcR) and an R6Kγ origin of replication for propagation in pir-dependent strains of Escherichia coli, as well as a hyperactive transposase for transposition of Tn-DS1028uidAKm. Locations of the transposon-specific primers PF294, PF1212, Nextera_i5_Adapt_Tn-specific_5F and Cust_Seq_Tn-specific_5F are shown. The image was prepared using Geneious R9 software [31]. (TIF) [file pone.0172790.s001.tif]

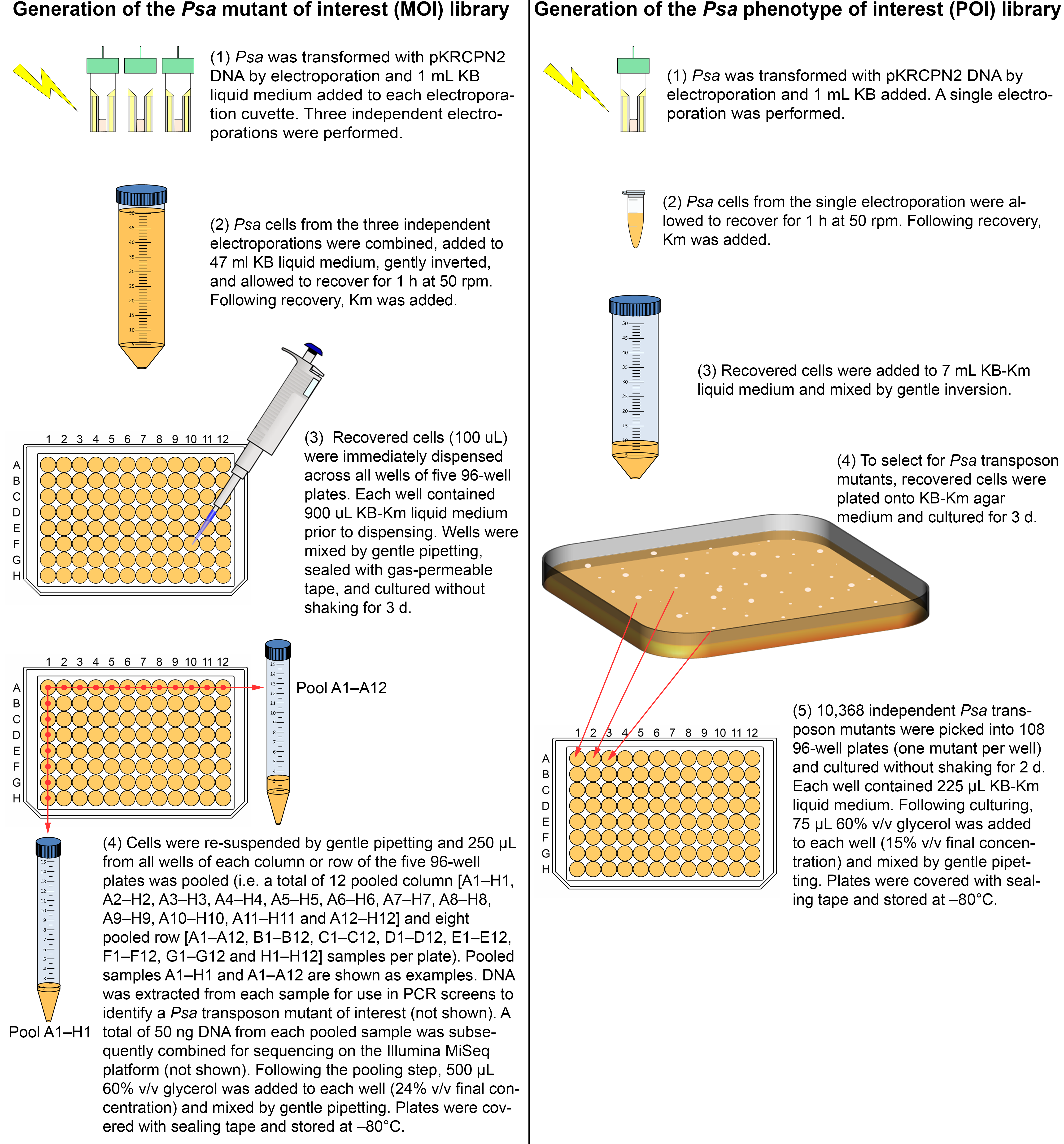

Supplement: S2 Fig — The final concentration of kanamycin (Km) used in King’s B (KB) agar and liquid media was 50 μg/mL. (TIF) [file pone.0172790.s002.tif]

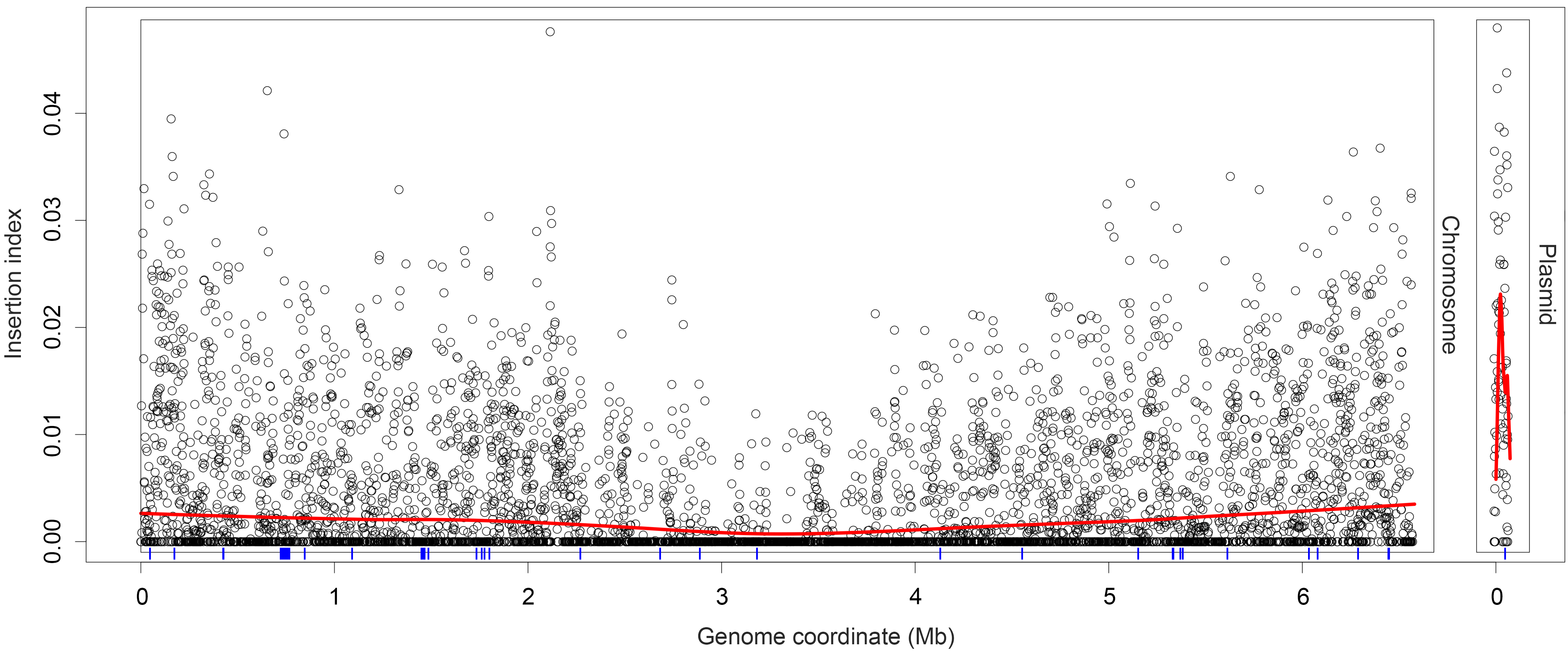

Supplement: S3 Fig — The scatter plots show the insertion index, computed for each protein-coding gene as a function of position on the Psa chromosome (left) and plasmid (right). In brief, the insertion index for each gene is computed using the count of unique transposon insertion sites in each gene, divided by the length of the gene. For example, a 108-bp length gene with 27 insertion sites has an insertion index of 27/108 = 0.25. A lowess curve (locally-weighted polynomial regression) is fitted to these two datasets, for the chromosome this shows a slight positional bias in the insertion indices. The genes near the origin of replication tend to have a higher insertion index, presumably because this region has a higher likelihood of receiving insertions during replication (two copies of the origin exist for longer than two copies of the terminus). The locations of Psa genes under the control of the HrpL regulon are shown by blue vertical dashes above the x axis. (TIF) [file pone.0172790.s003.tif]

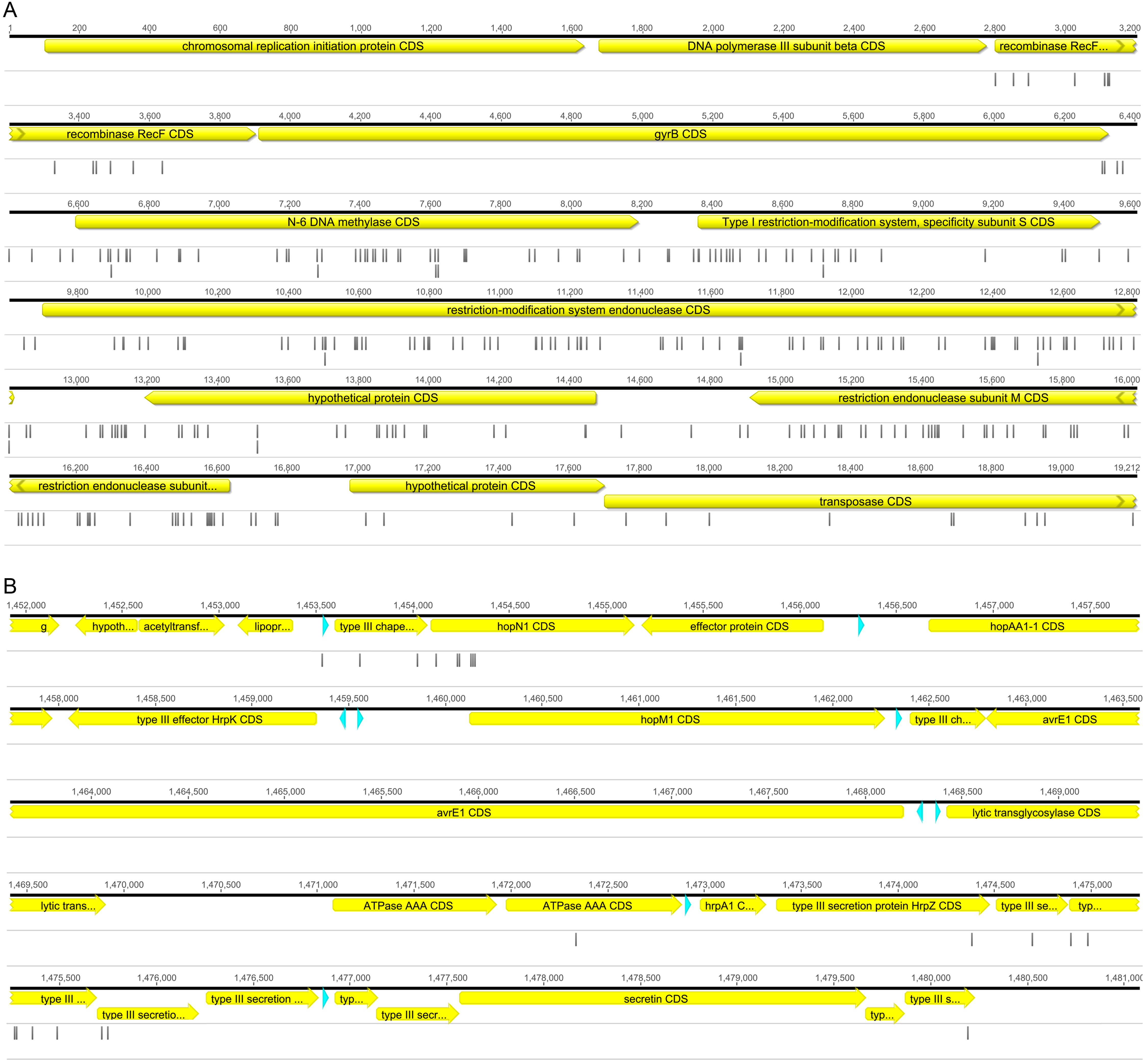

Supplement: S4 Fig — (A) a region of the Psa genome adjacent to the origin of replication; (B) a region from the conserved effector locus of the Psa genome. Transposon insertion sites are denoted by grey vertical lines, predicted coding regions as yellow bars and HrpL boxes by light blue triangles. The image was prepared using Geneious R9 software [31]. (TIF) [file pone.0172790.s004.tif]
