# Supplementary material for: Transposon insertion libraries for the characterization of mutants from the kiwifruit pathogen Pseudomonas syringae pv. actinidiae
Source: PLoS One. 2017 Mar 1;12(3):e0172790. doi: 10.1371/journal.pone.0172790 (PMC5332098; doi:10.1371/journal.pone.0172790)
Supplement: S1 Table — (DOCX) [file pone.0172790.s005.docx]

**Table S1. Primers used in this study.**

| Primer name | Primer sequence (5′–3′) | Primer description |
| --- | --- | --- |
| PF106^a^ | GACCACACGTCGACTAGTGCNNNNNNNNNNAGAG | Random-primed primer 1 for arbitrary PCR^b^ (first PCR) |
| PF107^a^ | GACCACACGTCGACTAGTGCNNNNNNNNNNACGCC | Random-primed primer 2 for arbitrary PCR (first PCR) |
| PF108^a^ | GACCACACGTCGACTAGTGCNNNNNNNNNNGATAC | Random-primed primer 3 for arbitrary PCR (first PCR) |
| PF294^a^ | CTTGCTCAATCAATCACCG | Tn-DS1028*uidA*Km-specific primer for arbitrary PCR (first PCR) |
| PF109^a^ | GACCACACGTCGACTAGTGC | Adapter primer for arbitrary PCR (second PCR) |
| PF1212 | CGGGAATTCTCATGTTTGAC | Nested Tn-DS1028*uidA*Km-specific primer for arbitrary PCR (second PCR). PCR screen forward primer for the identification of mutants with a disruption in the *GDP-6-deoxy-ᴅ-lyxo-4-hexulose reductase* gene |
| Nextera_i5_Adapt_Tn-specific_5F^c^ | AATGATACGGCGACCACCGAGATCTACACCGGGAATTCTCATGTTTGACAGCTTATCATC | Nextera i5 adaptor Tn-DS1028*uidA*Km-specific primer (library amplification) |
| Cust_Illumina_Nextera_i7_Adap_TruSeq_i5_Adap_V3.3_R^c^ | CAAGCAGAAGACGGCATACGAGATACACTCTTTCCCTACACGACGCTCTTCCGATCT | Custom Illumina Nextera i7 adapter Trueseq i5 adapter i5 V3.3 primer (library amplification) |
| Cust_Seq_Tn-specific_5F | TTTGACAGCTTATCATCGATAAGCTTGCCTCGCGCG | Nested custom sequencing primer (Illumina MiSeq sequencing) |
| Common_LPS_R6 | CTATCCCGCCAGTGGTGTC | PCR screen reverse primer for the identification of mutants with a disruption in the *GDP-6-deoxy-ᴅ-lyxo-4-hexulose reductase* gene |

^a^The PF106, PF107, PF108 and PF109 primers were taken from P. C. Fineran, *et al*. (1). The PF294 (also known as oTE55) primer was taken from T. J. Evans, *et al*. (2).

^b^PCR; Polymerase Chain Reaction.

^c^Oligonucleotide sequences © 2007-2012 Illumina, Inc. All rights reserved. Derivative works created by Illumina customers are authorized for use with Illumina instruments and products only. All other uses are strictly prohibited.

**References**

1. Fineran PC, Everson L, Slater H, Salmond GPC. A GntR family transcriptional regulator (PigT) controls gluconate-mediated repression and defines a new, independent pathway for regulation of the tripyrrole antibiotic, prodigiosin, in *Serratia*. Microbiology. 2005;151(12):3833-45.

2. Evans TJ, Ind A, Komitopoulou E, Salmond GP. Phage-selected lipopolysaccharide mutants of *Pectobacterium atrosepticum* exhibit different impacts on virulence. Journal of Applied Microbiology 2010;109(2):505-14.
