# Supplementary material for: Transposon insertion libraries for the characterization of mutants from the kiwifruit pathogen Pseudomonas syringae pv. actinidiae
Source: PLoS One. 2017 Mar 1;12(3):e0172790. doi: 10.1371/journal.pone.0172790 (PMC5332098; doi:10.1371/journal.pone.0172790)
Supplement: S2 Table — (DOCX) [file pone.0172790.s006.docx]

**Table S2. List of *Psa* transposon mutants identified in this study.**

| Mutant ID^a^ | Method of identification^b^ | Phenotype^c^ | Disrupted gene | Gene product | Gene size (bp) | Genome insertion site | Distance of insertion site from first bp of ATG gene start codon (bp) |
| --- | --- | --- | --- | --- | --- | --- | --- |
| APCR-1 | Arbitrary PCR screen | – | Intergenic | Between genes *IYO_029150* and *IYO_029155* | – | 6,500,633 | – |
| APCR-2 | Arbitrary PCR screen | – | *IYO_028735* | Membrane protein | 1,212 | 6,397,344 | 174 |
| APCR-3 | Arbitrary PCR screen | – | *IYO_029105* | Filamentous hemagglutinin/hemolysin | 8,835 | 6,487,770 | 5,770 |
| APCR-4 | Arbitrary PCR screen | – | Intergenic | Between genes *IYO_002620* and *IYO_002650* | – | 557,202 | – or 317 before ATG of *IYO_002650* |
| APCR-5 | Arbitrary PCR screen | – | *IYO_018825* | Integrase/mobile element protein | 954 | 4,200,587 | 657 |
| APCR-6 | Arbitrary PCR screen | – | *IYO_009880* | Flagellar basal-body rod modification protein FlgD | 690 | 2,119,266 | 567 |
| APCR-7 | Arbitrary PCR screen | – | *IYO_014615* | LuxR family DNA-binding transcriptional regulator | 639 | 3,237,468 | 100 |
| APCR-8 | Arbitrary PCR screen | – | *IYO_005420* | Transposase/mobile element protein | 1,431 | 1,157,070 | 569 |
| APCR-9 | Arbitrary PCR screen | – | *IYO_006240* | Glycosyl hydrolase | 7,782 | 1,350,663 | 250 |
| APCR-10 | Arbitrary PCR screen | – | *IYO_014250* | CheY-like receiver protein | 378 | 3,155,752 | 97 |
| APCR-11 | Arbitrary PCR screen | – | *IYO_001250* | ABC transporter substrate-binding protein | 1,884 | 260,673 | 1,352 |
| APCR-12 | Arbitrary PCR screen | – | *IYO_019475* | Soluble lytic murein transglycosylase | 1,929 | 4,357,306 | 827 |
| P95-A5 | POI auxotrophy screen | Auxotrophic mutant | *IYO_007710* | Enolase | 1,287 | 1,675,733 | 1,175 |
| P96-A9 | POI auxotrophy screen | Auxotrophic mutant | *IYO_007210* | Phosphoribosylformylglycinamidine synthase PurL | 3,897 | 1,557,755 | 1,736 |
| P96-B10 | POI auxotrophy screen | Auxotrophic mutant | *IYO_025960* | Dihydroxy-acid dehydratase IlvD | 1,848 | 5,780,845 | 1,114 |
| P96-G2 | POI auxotrophy screen | Auxotrophic mutant | *IYO_025885* | Dihydroorotase PyrC | 1,272 | 5,766,258 | 1,236 |
| P98-B6 | POI auxotrophy screen | Auxotrophic mutant | *IYO_008330* | Quinolinate synthetase NadA | 1,059 | 1,794,181 | 76 |
| P98-D7 | POI auxotrophy screen | Auxotrophic mutant | *IYO_008330* | Quinolinate synthetase NadA | 1,059 | 1,794,350 | 245 |
| P101-A9 | POI auxotrophy screen | Auxotrophic mutant | *IYO_007210* | Phosphoribosylformylglycinamidine synthase PurL | 3,897 | 1,557,195 | 1,176 |
| P101-F12 | POI auxotrophy screen | Auxotrophic mutant | *IYO_026305* | Glutamate synthase large subunit | 4,446 | 5,854,841 | 3,264 |
| P104-C2 | POI auxotrophy screen | Auxotrophic mutant | *IYO_012780* | 3-isopropylmalate dehydratase LeuD | 642 | 2,812,799 | 537 |
| P104-D2 | POI auxotrophy screen | Auxotrophic mutant | *IYO_008365* | Lipoprotein | 1,119 | 1,801,228 | 698 |
| P105-E10 | POI auxotrophy screen | Auxotrophic mutant | *IYO_008720* | Phosphoribosylglycinamide formyltransferase PurN | 651 | 1,876,398 | 250 |
| P106-A4 | POI auxotrophy screen | Auxotrophic mutant | *IYO_027710* | Histidine kinase | 867 | 6,176,215 | 17 |
| P106-C11 | POI auxotrophy screen | Auxotrophic mutant | *IYO_006345* | Phosphogluconate dehydratase | 1,827 | 1,374,151 | 186 |
| P106-E7 | POI auxotrophy screen | Auxotrophic mutant | *IYO_006370* | Sugar ABC transporter permease | 909 | 1,381,444 | 873 |
| P106-G7 | POI auxotrophy screen | Auxotrophic mutant | Intergenic or *IYO_025960* | Between genes *025960* and *025965* or promoter of dihydroxy-acid dehydratase IlvD | – | 5,782,023 | – or 125 before ATG |
| P106-H6 | POI auxotrophy screen | Auxotrophic mutant | *IYO_001755* | 2,3-bisphosphoglycerate-independent phosphoglycerate mutase | 1,533 | 355,027 | 44 |
| P108-G11 | POI auxotrophy screen | Auxotrophic mutant | *IYO_008720* | Phosphoribosylglycinamide formyltransferase PurN | 651 | 1,876,432 | 216 |
| P62-E3 | POI swimming screen | Non-swimming mutant | *IYO_010080* | Flagellar biosynthesis protein FliR | 777 | 2,164,764 | 259 |
| P64-C6 | POI swimming screen | Non-swimming mutant | *IYO_009875* | Flagellar basal-body rod protein FlgC | 441 | 2,118,499 | 256 |
| P65-B11 | POI swimming screen | Non-swimming mutant | *IYO_009930* | FlgL/flagellar hook-associated protein FlgL | 1,593 | 2,130,249 | 243 |
| P72-E3 | POI swimming screen | Non-swimming mutant | *IYO_010090* | Flagellar biosynthesis protein FlhA | 2,130 | 2,168,037 | 1,240 |
| P61-B4 | POI swimming screen | Super-swimming mutant | *IYO_020475* | Acetate permease ActP | 1,659 | 4,567,897 | 291 |
| P62-D5 | POI swimming screen | Super-swimming mutant | *IYO_024315* | Membrane protein TraG | 1,539 | 5,405,222 | 960 |
| P60-C4 | POI swimming screen | Weak-swimming mutant | *IYO_007210* | Phosphoribosylformylglycinamidine synthase PurL | 3,897 | 1,559,351 | 3,332 |
| P60-E7 | POI swimming screen | Weak-swimming mutant | *IYO_026350* | Transpeptidase-transglycosylase | 2,445 | 5,867,762 | 730 |
| P1-A1 | Visual screen | LPS mutant (common LPS biosynthesis pathway) | *IYO_023030* | Glycosyl transferase | 1,110 | 5,120,107 | 379 |
| P1-A2 | Visual screen | LPS mutant (core LPS biosynthesis pathway) | *IYO_025555* | Membrane protein | 1,176 | 5,673,161 | 88 |
| P1-A3 | Visual screen | LPS mutant (unannotated LPS gene?) | *IYO_008995* | Glycosyl transferase | 1,017 | 1,937,028 | 814 |
| P1-A4 | Visual screen | LPS mutant (common LPS biosynthesis pathway) | *IYO_023015* | ABC transporter permease RfbD | 795 | 5,116,883 | 269 |
| P1-A6 | Visual screen | LPS mutant (common LPS biosynthesis pathway) | *IYO_023005* | SAM-dependent methyltransferase WbbD | 1,548 | 5,114,421 | 726 |
| P1-A12 | Visual screen | N/A | *IYO_018250* | Glycerol-3-phosphate dehydrogenase | 1,026 | 4,057,170 | 80 |
| P1-B4 | Visual screen | LPS mutant (common LPS biosynthesis pathway) | *IYO_023010* | ABC transporter ATP-binding protein | 1,218 | 5,116,245 | 116 |
| P1-B5 | Visual screen | LPS mutant (unannotated LPS gene?) | *IYO_005330* | Glycosyl transferase | 1,149 | 1,139,578 | 379 |
| P1-B6 | Visual screen | LPS mutant (common LPS biosynthesis pathway) | *IYO_023005* | SAM-dependent methyltransferase WbbD (different transposon insertion site than that found in mutant A6) | 1,548 | 5,113,975 | 1,172 |
| P1-B12 | Visual screen | LPS mutant (common LPS biosynthesis pathway) | *IYO_023020* | GDP-mannose 4,6-dehydratase | 1,032 | 5,117,719 | 36 |
| P1-C2 | Visual screen | N/A | *IYO_009915* | Flagellar P-ring protein Flgl | 1,110 | 2,125,869 | 319 |
| P1-X10 | LPS MOI screen | LPS mutant (common LPS biosynthesis pathway)? | *IYO_023025* | GDP-6-deoxy-ᴅ-lyxo-4-hexulose reductase | 897 | 5,118,839 | 125 |
| P1-DX | LPS MOI screen | LPS mutant (common LPS biosynthesis pathway)? | *IYO_023025* | GDP-6-deoxy-ᴅ-lyxo-4-hexulose reductase | 897 | 5,118,891 | 177 |
| P1-G6(H5) | LPS MOI screen | LPS mutant (common LPS biosynthesis pathway)? | *IYO_023025* | GDP-6-deoxy-ᴅ-lyxo-4-hexulose reductase | 897 | 5,119,352 | 638 |
| P2-B3 | LPS MOI screen | LPS mutant (common LPS biosynthesis pathway)? | *IYO_023025* | GDP-6-deoxy-ᴅ-lyxo-4-hexulose reductase | 897 | 5,118,931 | 217 |
| P2-C5 | LPS MOI screen | LPS mutant (common LPS biosynthesis pathway)? | *IYO_023025* | GDP-6-deoxy-ᴅ-lyxo-4-hexulose reductase | 897 | 5,119,449 | 735 |
| P3-D4 | LPS MOI screen | LPS mutant (common LPS biosynthesis pathway)? | *IYO_023025* | GDP-6-deoxy-ᴅ-lyxo-4-hexulose reductase | 897 | 5,119,429 | 715 |
| P3-G10 | LPS MOI screen | LPS mutant (common LPS biosynthesis pathway)? | *IYO_023025* | GDP-6-deoxy-ᴅ-lyxo-4-hexulose reductase | 897 | 5,118,793 | 79 |
| P4-G12 | LPS MOI screen | LPS mutant (common LPS biosynthesis pathway)? | *IYO_023025* | GDP-6-deoxy-ᴅ-lyxo-4-hexulose reductase | 897 | 5,119,461 | 747 |
| P4-H5 | LPS MOI screen | LPS mutant (common LPS biosynthesis pathway)? | *IYO_023025* | GDP-6-deoxy-ᴅ-lyxo-4-hexulose reductase | 897 | 5,119,476 | 762 |
| P1-X5 | LPS MOI screen | – | *IYO_020495* | ABC transporter | 813 | 4,572,177 | 13 |

^a^Mutants APCR-1–12, P1-X10, P1-DX, P1-G6(H5), P2-B3, P2-C5, P4-G12, P4-H5 and P1-X5 were not stored as glycerol stocks. X; column or row is unknown due to absence of PCR amplicon DNA sequence information.

^b^PCR; polymerase chain reaction, POI; phenotype of interest, MOI; mutant of interest.

^c^LPS; lipopolysaccharide, N/A; not applicable.
