# Supplementary material for: Transposon insertion libraries for the characterization of mutants from the kiwifruit pathogen Pseudomonas syringae pv. actinidiae
Source: PLoS One. 2017 Mar 1;12(3):e0172790. doi: 10.1371/journal.pone.0172790 (PMC5332098; doi:10.1371/journal.pone.0172790)
Supplement: S3 Table — (DOCX) [file pone.0172790.s007.docx]

**Table S3. Transposon insertion settings and statistics.**

| Setting | Chromosome | | | Plasmid | | |
| --- | --- | --- | --- | --- | --- | --- |
|  | **Total independent insertion sites** | **Average bp/insert^a^** | **Inserts/kb^b^** | **Total independent insertion sites** | **Average bp/insert** | **Inserts/kb** |
| ‘Sensitive’ | 84,334 | 78.0 | 12.8 | 3,041 | 24.5 | 40.9 |
| ‘Default’ | 50,990 | 129 | 7.75 | N/A^c^ | N/A | N/A |
| ‘Specific’ | 24,031 | 274 | 3.65 | 1,236 | 60.2 | 16.6 |

^a^bp, base pairs.

^a^kb, kilobases.

^c^N/A, not available.
