# Supplementary material for: Transposon insertion libraries for the characterization of mutants from the kiwifruit pathogen Pseudomonas syringae pv. actinidiae
Source: PLoS One. 2017 Mar 1;12(3):e0172790. doi: 10.1371/journal.pone.0172790 (PMC5332098; doi:10.1371/journal.pone.0172790)
Supplement: S1 Methods — (DOCX) [file pone.0172790.s008.docx]

**Supplementary Methods**

Arbitrary PCR

PCR 1 setup:

| Reagent | Volume (µl) |
| --- | --- |
| 10× PCR Buffer, minus Mg (Invitrogen, Carlsbad, CA, USA)  50 mM MgCl_2_ (Invitrogen) | 2.00  0.60 |
| 10 mM dNTPs (Invitrogen) | 0.40 |
| 10 µM PF294 | 1.25 |
| 10 µM PF106 | 0.35 |
| 10 µM PF107 | 0.35 |
| 10 µM PF108 | 0.35 |
| Platinum *Taq* DNA Polymerase (Invitrogen) | 0.20 |
| 1 ng/µl DNA template | 2.00 |
| Autoclaved, distilled water | 12.5 |
| Total | 20.00 |

PCR 1 program:

Set 1 (×1)

Denaturation 94°C for 3 min

Set 2 (×6)

Denaturation 94°C for 15 sec

Annealing 42°C for 30 sec

(+1 °C / cycle)

Elongation 72°C for 3 min

Set 3 (×25)

Denaturation 94°C for 15 sec

Annealing 47.9°C for 30 sec

Elongation 72°C for 3 min

Set 4 (×1)

Final elongation 72°C for 7 min

Hold 12°C forever

PCR 2 setup:

| Reagent | Volume (µl) |
| --- | --- |
| 10× PCR Buffer, minus Mg | 5.00 |
| 50 mM MgCl_2_  10 mM dNTPs | 1.50  1.00 |
| 10 µM PF1212 | 3.125 |
| 10 µM PF109 | 3.125 |
| Platinum *Taq* DNA Polymerase | 0.50 |
| PCR 1 DNA template (1:10 dilution) | 5.00 |
| Autoclaved, distilled water | 30.75 |
| Total | 50.00 |

PCR 2 program:

Set 1 (×1)

Denaturation 94°C for 3 min

Set 2 (×35)

Denaturation 94°C for 15 sec

Annealing 47.9°C for 30 sec

Elongation 72 °C for 3 min

Set 3 (×1)

Final elongation 72°C for 7 min

Finish
